# The herbal decoction modified Danggui Buxue Tang attenuates immune-mediated bone marrow failure by regulating the differentiation of T lymphocytes in an immune-induced aplastic anemia mouse model

**DOI:** 10.1371/journal.pone.0180417

**Published:** 2017-07-06

**Authors:** Peiying Deng, Xue Li, Yi Wei, Juan Liu, Meng Chen, Yamei Xu, Bin Dong, Lingqun Zhu, Limin Chai

**Affiliations:** 1 Key Laboratory of Chinese Internal Medicine of Ministry of Education and Beijing, Dongzhimen Hospital, Beijing University of Chinese Medicine, Beijing, China; 2 Department of Hematology & Oncology, Dongzhimen Hospital, Beijing University of Chinese Medicine, Beijing, China; 3 School of Preclinical Medicine, Beijing University of Chinese Medicine, Beijing, China; Stavanger University Hospital, NORWAY

## Abstract

*Angelicae Sinensis*, *Radix Astragali* and *Rhizoma Coptidis* are all herbs of modified Danggui Buxue Tang (DGBX) and are extensively applied herbs in traditional Chinese medicine for the treatment of anemia and inflammation. In this study, immune-induced AA mice were used as an animal model, and the immunosuppressive agent, Ciclosporin A (CsA), was used as a positive control. Multiple pro-inflammatory cytokines were examined by bead-based multiplex flow cytometry. The T-cell subsets were assessed using a fluorescence-activated cell sorter (FACS). Western blot analysis was used to estimate the protein expression levels of specific transcription factors for T helper cells (Th1, Th2 and Th17) and key molecules of the Janus-activated kinase (Jak)/signal transducer and activator of transcription (Stat3) signaling pathway. DGBX treatment could significantly increase the production of whole blood cells in peripheral blood (PB); inhibit the expansion of Th1 and Th17 cells; increase the differentiation of Th2 and Tregs cells; regulate the expression levels of T-bet, GATA-3, RORγ and proinflammatory cytokines; and decrease the expression levels of key molecules in the Jak/Stat signaling pathway. These results indicate that DGBX can regulate the differentiation of T lymphocytes, resulting in immunosuppressive and hematogenic functions on AA mice. DGBX might be a good candidate for inclusion in a randomized study for AA with more data on the possible side effects and doses used in humans. Ultimately, it may be used for applications of traditional medicine against AA in modern complementary and alternative immunosuppressive therapeutics.

## Introduction

Aplastic anemia (AA) is a bone marrow failure syndrome characterized by the reduction or absence of mature hemopoietic progenitors in all cell lineages [1]. Most cases of AA are associated with an aberrant immune response. Cytotoxic T cells (CD8^+^) are expanded in AA and are involved in the production of proinflammatory cytokines, including interferon (IFN) γ, tumor necrosis factor (TNF) αand interleukin (IL)-1β, which induce immune destruction and apoptosis of hematopoietic progenitor cells [2]. Several studies have confirmed that T helper (Th) cells, including IFNγ-producing (Th1), IL-4-producing and IL-17-producing (Th17) CD4^+^ T cells, play pivotal roles in autoimmunity [3, 4] and contribute to the pathogenesis of AA. Regulatory T cells (Tregs), specifically expressing CD25 and transcription factor FOXP3, maintain the immunologic self-tolerance and immunosuppression [5, 6]. Intrinsic impairment of Tregs plays a critical role in the pathophysiology of AA [7]. Treg-mediated immunosuppressive strategies should contribute to suppressing excessive Th1 and Th17 immune responses in AA.

In traditional Chinese medicine (TCM), herbal decoctions are specific combinations of different herbs that are used as formulas for unique methods [8]. Combinatory therapeutic strategies use multiple herbal decoctions based on the patient’s symptoms and characteristics and are used to treat several diseases [9]. Multiple components act on multiple targets and exert synergistic therapeutic effects [10]. *Radix Angelicae Sinensis*, *Radix Astragali* and *Rhizoma Coptidis* are the extensively applied herbs in TCM used for the treatment of anemia and inflammation. A modified herbal decoction, DGBX, is composed of these three herbal medicines. It is derived from a famous Chinese herbal decoction Danggui Buxue Tang (DBT). The pharmacological properties of DBT have been illustrated in hematopoiesis, thrombopoiesis and immune regulation [11–13]. *Rhizoma coptidis* is well known for its anti-inflammatory, antioxidative, antiviral, and antimicrobial functions. Pharmacological studies have shown that *Rhizoma coptidis* can significantly protect mice against LPS-induced acute liver injury [14] and inhibit LPS-induced MCP-1/CCL2 production in vitro in an AP-1- and NF-κB-dependent manner [15]. It also has an anticachectic effect on esophageal cancer, and an effect associated with the ability of berberine to down-regulate tumor IL-6 production [16]. In our previous studies, DGBX (also called Bushen Shengxue Jiedu Fang) was found to promote the activition of stem cell factors, induce the proliferation and differentiation of hematopoietic stem cells (HSCs), inhibit immune reactions induced by IFNγ, and recover the normal functions of HSCs [17, 18]. However, the molecular mechanism by which DGBX affected the proliferation and differentiation of T lymphocytes for immunosuppresion was unclear.

Here, we studied the potential molecular mechanisms of immunosuppressive and hematopoietic functions of DGBX in immune-mediated AA. The expression levels of key molecular molecules of Janus-activated kinase (Jak)/signal transducer and activator of transcription (Stat) were assessed. We wanted to identify the specific cellular and protein targets involved in the immunosuppressive function of DGBX on AA treatment.

## Materials and methods

### Preparation of the herbal composition of DGBX

A total of 126 g of raw herbal pieces, including *Radix Astragali* (origin of inner Mongolia, China, 90 g), *Radix Angelicae Sinensis* (origin of Gansu, China, 18 g) and *Coptis chinensis Franch* (origin of Sichuan, China, 18 g) (individual ratio = 5:1:1, according to usual clinical dosage), were first boiled together in a 6× volume of water for 30 min. The residue from the first extraction was boiled in an 8× volume of water for 25 min. Finally, the filtered solutions were combined and concentrated into a volume of 140 mL. One milliliter of aqueous extract solution contains 0.9 g/mL raw herbs. The raw herbal pieces were purchased from Beijing Xidan Pharmaceutical Co., Ltd., China.

### High-performance liquid chromatography-mass spectrometer (HPLC-MS) analysis

The herbal extract was filtered using a standard test sieve of 150 μm, freeze-dried and maintained in desiccators at 4°C until use. Next, 0.02 g of the lyophilized powder was extracted with 5 mL methanol/water (v/v = 1:1) in the sonicator for 20 min at room temperature before being filtered through a 0.22-μm membrane. A 10 μL aliquot of the extract was injected into the analytical column for analysis. The separation of the compounds was carried out on a Phenomenex Kinetex C18 (2.6 μm, 100 × 2.1 mm) operated at 35°C. The mobile phase, which consists of 0.1% formic acid in water (A) and acetonitrile (B), was delivered at a flow rate of 0.4 mL/min under a gradient program. The diode-array detector was set to monitor at 254 nm, and the online UV spectra were recorded in the scanning range of 190–400 nm. The mass spectra were acquired using a TripleTOF™ 4600 system with a Duo Spray source (SCIEX, Foster City, CA, USA) in negative and positive ESI mode. For TOF-MS and TOF-MS/MS analysis, the spectra covered the range from m/z 100 to 1,000 Da and 50–1000 Da. The data were analyzed by Peak View Software™ 2.2 (SCIEX, Foster City, CA, USA). The substances were identified by HPLC-MS based on retention time, accurate mass and MS/MS spectrum comparison to spectra available in the public literature.

### Mice

BALB/c female mice (n = 6 per group, 7 to 8 weeks old) and DBA/2 female mice (7 to 8 weeks old) were purchased from HFK Bioscience Co. Ltd. (Beijing, China). Animal care and use were conducted in accordance with institutional guidelines, and all animal experiments were approved by the Institutional Animal Care and Use Committee of the National Institute of State Scientific and Technological Commission.

### Induction of AA

The method for inducing the AA mouse model was as follows. BALB/c mice, except for mice in the normal group, received a sublethal total body irradiation dose of 3.5 Gy from Model 143 ^137^Cesium μ-irradiator one hour before lymph node cell infusion. Inguinal, brachial and axillary infusion of lymph node cells was obtained from female DBA/2 mice. A total of 1×10^6^ lymph cells were infused into the BALB/c mice by intravenous tail injections to induce AA [19, 20]. Mice in the normal group were irradiated with lead brick and injected with an isodose of physiological saline.

### Drug treatment

Drug treatment was started after lymph node cell infusion and lasted for 28 days. Mice were randomly divided into four groups as follows. In the normal group, mice were fed the control diet and orally given sterile saline. In the model group, mice were fed the same as the normal group. In the Cyclosporin (CsA) group, CsA (Batch No. H10960122, Zhongmei Huadong Pharmaceutical Co. LTD, Hangzhou, China) was provided to mice fed the same control diet. These mice were orally given 25 mg/kg CsA daily for the 28-day experimental period. In the DGBX group, mice were fed the same control diet and orally given 6.3 g/kg DGBX (equivalent to the human dose) daily for the 28-day experimental period. The mice were sacrificed on the 29th day after treatment. Mice were anesthetized by isoflurane anesthesia (2–3% isoflurane with oxygen supply). Peripheral blood (PB) was obtained by removing the eyeballs, while bone marrow cells (BMCs) were obtained by femoral cavity flushing.

### Whole blood cell analysis

Whole blood cell analysis was examined on days 7, 14, 21 and 28 of the experimental period. Ten microliters of whole blood was obtained by cutting the mouse tail tip. Blood cells were suspended in 2 mL PBS buffer for analysis. Cell analysis was executed using the Blood Cell Analyzer (Nihon Kohden, Tokyo, Japan), according to the manufacturer’s instruction manual. Red blood cells (RBCs), white blood cells (WBCs), hemoglobin (HB) and platelets (PLTs) were quantified.

### Bead-based multiplex flow cytometry

A total of 0.8 mL of PB was collected from each mouse by eyeball extirpation 24 hours after the last administration. Blood samples were incubated at room temperature for 60 min, and sera were isolated by centrifugation at 500 g and 4°C for 10 min. Aimplex Mouse Inflammation 6-Plex assay kits (Quantobio, Beijing China), including IFNγ, IL-2, IL-4, IL-6, IL-10 and IL-17a, were used according to the manufacturer’s instruction manual. Briefly, the assay procedure consisted of a 60-min antigen and capture-antibody-conjugated bead incubation step, a 30-min biotinylated detection incubation step and a 20-min streptavidin-PE incubation step. Fluorescence signals of the sample beads were acquired by a flow cytometer, and the inflammatory cytokines were analyzed using FCAP Array 3.0 (BD Biosciences, NYC, NY, USA).

### Fluorescence-activated cell sorter (FACS) analysis

Half of the spleens were removed, diced, and expressed through a 40-μm Nylon mesh. Then, 0.4 mL of peripheral blood was collected from each mouse and suspended in 0.6 mL PBS buffer. To quantify the percentages of CD3^+^CD4^+^ and CD3^+^CD8^+^ cells, cells were washed and stained with anti-mouse CD3 PE-Cyanine7, CD4 PE-Cyanine5 and CD8a PE antibodies (eBioscience, San Diego, CA, USA). To determine the percentages of Th1, Th2 or Th17 cells, splenocytes were stimulated with phorbol 12-myristate 13-acetate (PMA) (50 ng/ml) and ionomycin (Ion) (1 μg/ml) (Sigma, San Francisco, CA, USA) for 5 h in the presence of GolgiPlug (BD Bioscience) according to the manufacturer’s protocol. Cells were washed and stained with anti-mouse CD4 PE-Cyanine5 antibody (eBioscience). Following CD4 staining, cells were blocked, fixed and permeabilized using the Fixation/Permeabilization kit according to the manufacturers’ instructions (BD Bioscience). They were then stained with anti-mouse IFNγ-PE, IL-4 PE-Cyanine7 or anti-mouse/rat IL-17a FITC antibodies (BD Bioscience). To determine the percentages of Treg cells, cells were stained with anti-mouse CD4 PE-Cyanine5 and anti-mouse CD25 PE antibodies (eBioscience). Following the CD4 and CD25 staining, cells were blocked, fixed and permeabilized using the Fixation/Permeabilization kit according to the manufacturers’ instructions (BD Bioscience), and stained with anti-mouse/rat-FOXP3- FITC antibody (eBioscience). Flow cytometry was performed by a FACS Calibur cytometer and analyzed by CellQuest software (Beckman Coulter, Brea, CA, USA).

### Western blot analysis

The other half of the spleen of each mouse was lysed in 0.5 mL of a lysis buffer (Sigma). The extracts were cleared by centrifugation at 10,000 g at 4°C for 15 minutes and then diluted with lysis buffer to achieve approximately 2 mg/mL protein concentration. Protein samples were separated on 10% SDS-PAGE and transferred onto nitrocellulose membranes (Amersham Pharmacia Biotech, Uppsala, Sweden). The membranes were incubated with primary antibodies, including anti-T-bet/Tbx21 antibody (1:1000), (Abcam, Cambridge, MA, USA), anti-mouse GATA-3, RORγ, Jak1, Stat-1 and Stat-3 rabbit monoclonal antibodies (CST, Boston, MA, USA), and were then incubated with a horseradish peroxidase-conjugated secondary antibody (CST). All immunoreactive proteins were visualized with SuperSignals west Pico Chemiluminescent Substrate (Thermo Scientific, Rockford, IL, USA). Densitometry plots showing the protein expression levels were normalized to GAPDH and expressed in terms of the fold change relative to the levels in the normal group.

### Statistical analysis

All data are presented as the mean ± standard deviation (S.D.). The statistical analyses were performed using SPSS13.0 (SPSS Inc., Chicago, IL, USA). One-way analyses of variance (ANOVA) followed by the Tukey-Kramer test for multiple comparisons was used to compare the treatment groups. *P*<0.05 was considered to be statistically significant.

## Results

### Identification of the chemical constituents in DGBX by HPLC-MS

**Fig 1:**
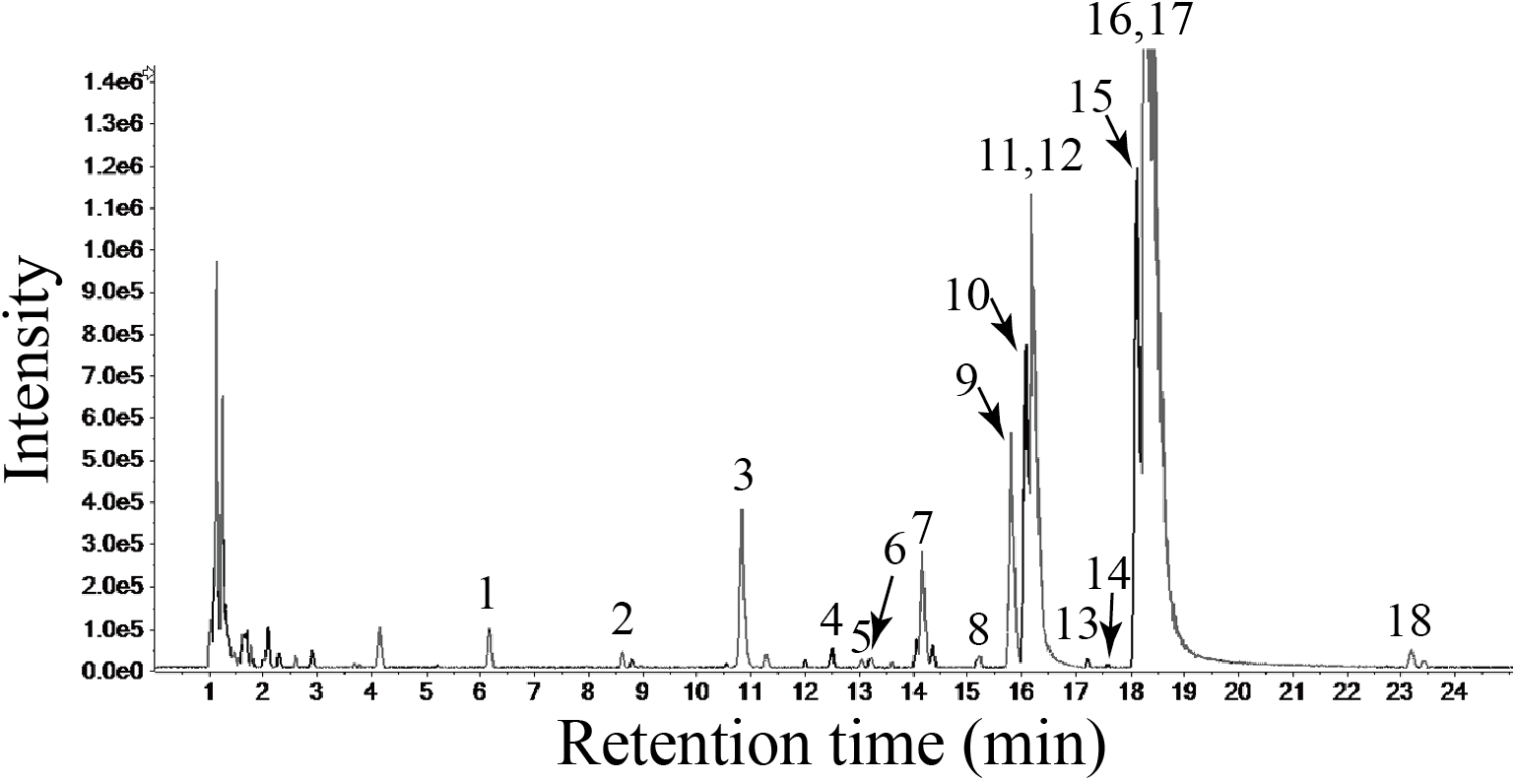
HPLC-MS Base Peak Chromatogram (BPC) of the total ion chromatogram (TIC) for the lyophilized powder of DGBX water decoction. The abscissa represents the retention time, while the ordinate represents the chromatographic peak intensity.

Representative liquid chromatography-mass spectrometry chromatograms are shown in Fig 1. Eighteen constituents were identified by the accurate mass and relative ion abundance of the target peaks. The predominant constituents in DGBX identified through the target peaks were epiberberine (11), coptisine (12), berberine (16) and calycosin (17). The secondary constituents were magnoflorine (3), jatrorrhizine (9), ononin (10) and palmatine (15). L-tryptophan (1), feruloylquinic (2), calycosin-7-O-β-D-glucoside (4), ferulic acid (5), tetradehydroscoulerine (7) and Calycosin-7-O-β-D-glucoside-6''-O-malonate (8) were also identified from the lyophilized powder of DGBX aqueous extracts. The area percentage for each of the constituents is shown in Table 1.

**Table 1:** The area percentage for each of the constituents in lyophilized powder.

No.	Retention time	Constituent	Peak Area	Area %
1	6.14	L-tryptophan	5440.812553	2.0432
2	8.61	3-O-Feruloylquinic	4588.19873	1.7230
3	10.82	Magnoflorine	13382.70745	5.0256
4	12.5	Calycosin-7-O-β-D-glucoside	3992.375891	1.4993
5	13.04	Ferulic acid	4009.576298	1.5057
6	13.2	Senkyunolide I	1309.902567	0.4919
7	14.17	tetradehydroscoulerine	3814.129456	1.4323
8	15.22	Calycosin-7-O-β-D-glucoside-6''-O-malonate	3594.518283	1.3498
9	15.81	Jatrorrhizine	9032.289494	3.3919
10	16.01	Ononin	15605.47807	5.8603
11	16.08	epiberberine	49569.11554	18.6147
12	16.21	Coptisine		
13	16.67	(6aR,-11aR)-3-Hydroxy-9,10- dimethoxypterocarpan-3-O-β-D-glucoside	296.8966796	0.1115
14	17.6	Z-Butylidenephthalide	131.5811972	0.0494
15	18.12	Palmatine	23821.41377	8.9456
16	18.31	Berberine	127700.1708	47.9552
17	18.47	Calycosin	0.439904637	0.0002
18	23.19	Formononetin	0.994467535	0.0004

### DGBX improved hemocytopoiseis in the AA mouse model

**Fig 2:**
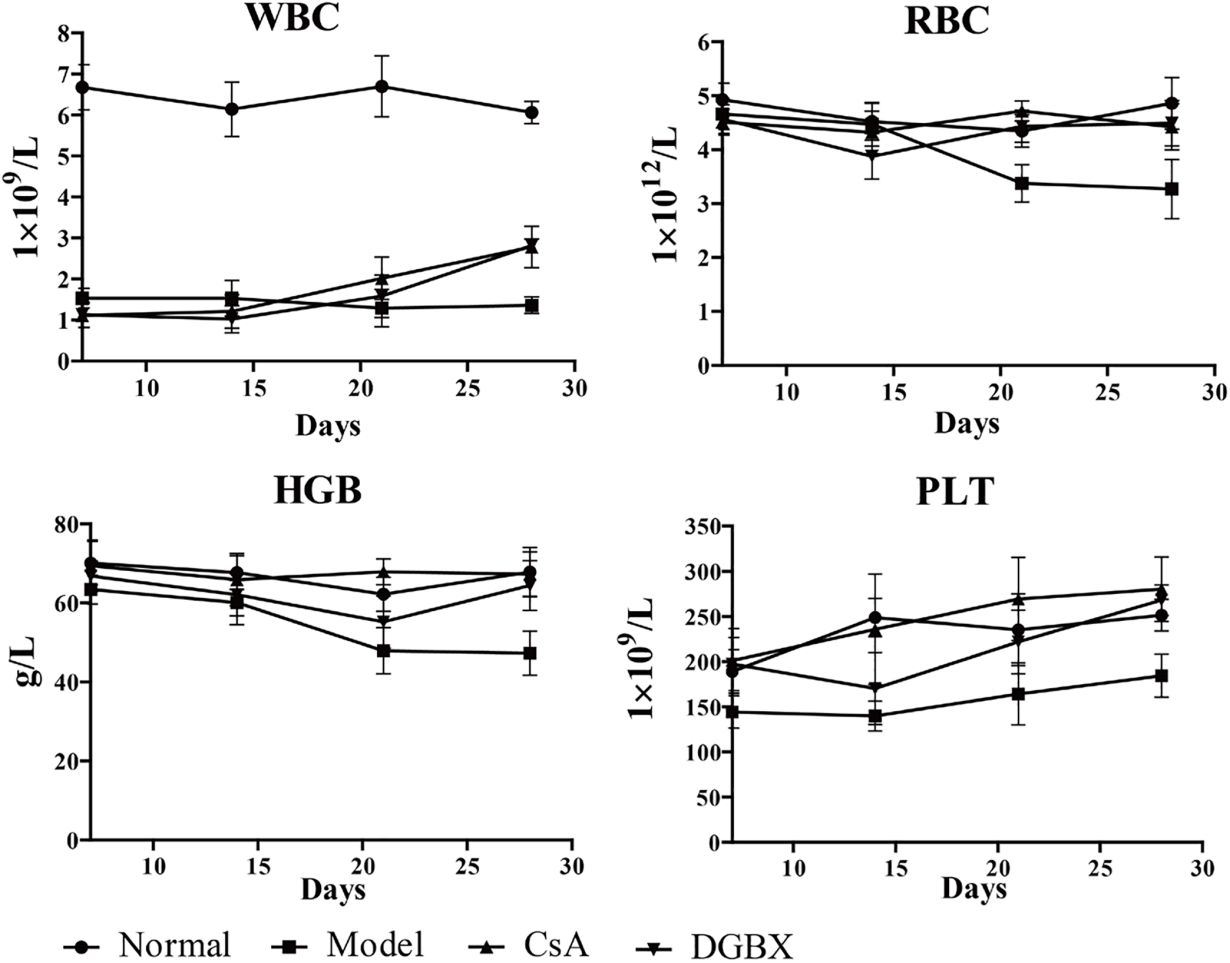
Effects of DGBX on hemocytopoiseis in AA mice. RBC, red blood cell; WBC, white blood cell; HGB, hemoglobin; PLT, platelet.

To assess the hematopoiesis function of DGBX on AA, whole blood cell analysis was performed in the AA mouse model. The quantities of WBCs and PLTs in PB of AA mice were decreased significantly compared to those of normal mice on the 7th day after inducing the AA model, and the quantities RBCs and HGB were reduced significantly on the 21st day after making the model. Treatment by DGBX and CsA increased the quantities of whole blood cells in PB significantly at 14–28 days after AA mouse model induction (Fig 2).

### Effect of DGBX on the proliferation and differentiation of CD3^+^ T lymphocytes in the AA mouse model

**Fig 3:**
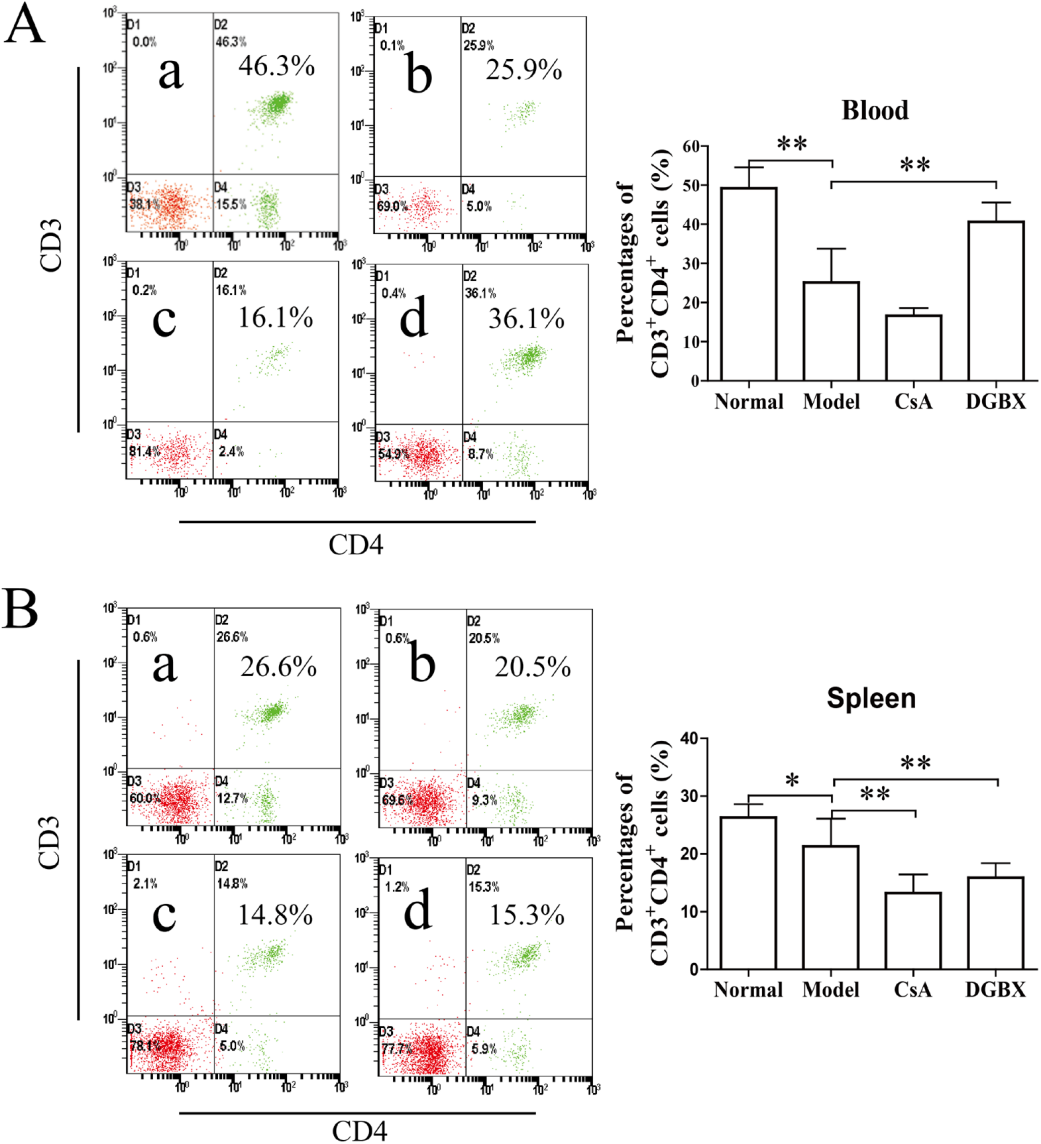
DGBX regulates the proliferation of CD3^+^CD4^+^ T cells in PBs and splenocytes. (A) The percentages of CD3^+^CD4^+^ T cells in PBs of mice after 4 weeks of treatment. (B) The percentages of CD3^+^CD4^+^ T cells in splenocytes of mice after 4 weeks of treatment. The results are presented in the bar charts; a. normal group, b. model group, c. group treated with CsA, and d. group treated with DGBX. The data are presented as means ± SD, n = 6. *P<0.05, **P < 0.01.

**Fig 4:**
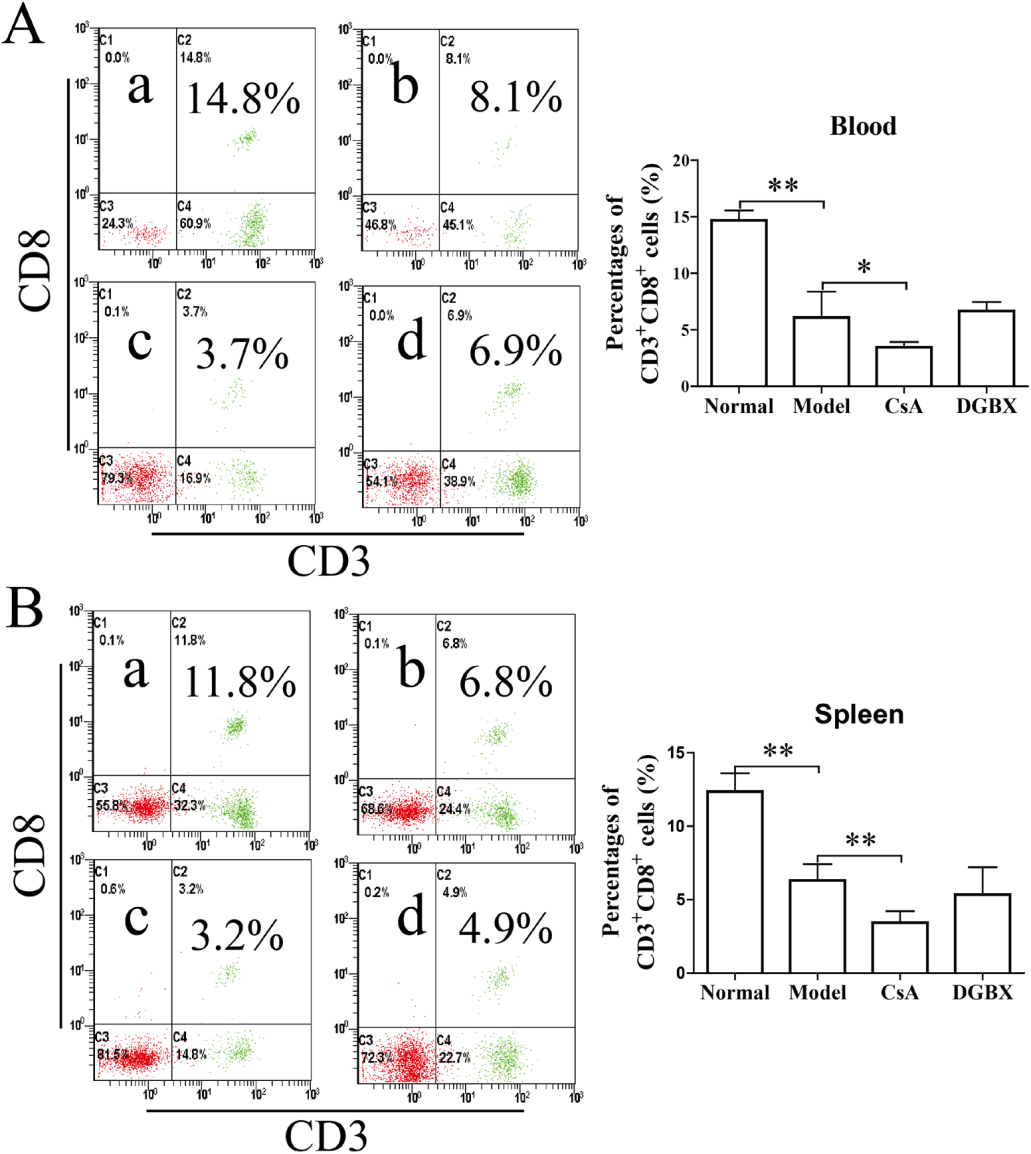
DGBX regulates the proliferation of CD3^+^CD8^+^ cytotoxic T cells (CTLs) in PBs and splenocytes. (A) The percentages of CD3^+^CD8^+^ CTLs in PBs of mice after 4 weeks of treatment. (B) The percentages of CD3^+^CD4^+^ CTLs in splenocytes of mice after 4 weeks of treatment. The results are presented in the bar charts; a. normal group, b. model group, c. group treated with CsA, and d. group treated with DGBX. Data are presented as means ± SD, n = 6. *P<0.05, **P < 0.01.

AA is an immune-mediated disorder characterized by the active destruction of hematopoietic cells by effector T lymphocytes [21, 22]. We measured the percentages of CD3^+^CD4^+^ cells and CD3^+^CD8^+^ cells in PBs and splenocytes of AA mice after 28 days of treatment. The percentages of CD3^+^CD4^+^ cells of AA mice were significantly reduced compared with those of normal mice (*P*<0.01 PB, *P*<0.05 spleen). The level of the CsA treatment group was significantly decreased (*P*<0.01, compared with the model group). Strangely, this percentage in the PB of the DGBX group was maintained at a higher level after treatment (Fig 3). The percentages of cytotoxic T cells (CD3^+^CD8^+^) in the PB and spleen of AA mice were also significantly decreased compared to those of normal mice (*P*<0.01). The level of cytotoxic T cells in the AA mice treated by CsA was lower than that in other groups (*P*<0.01 or *P*<0.05) (Fig 4).

### DGBX regulates the differentiation of CD4^+^CD25^+^FOXP3^+^ regulatory T cells in AA mice

**Fig 5:**
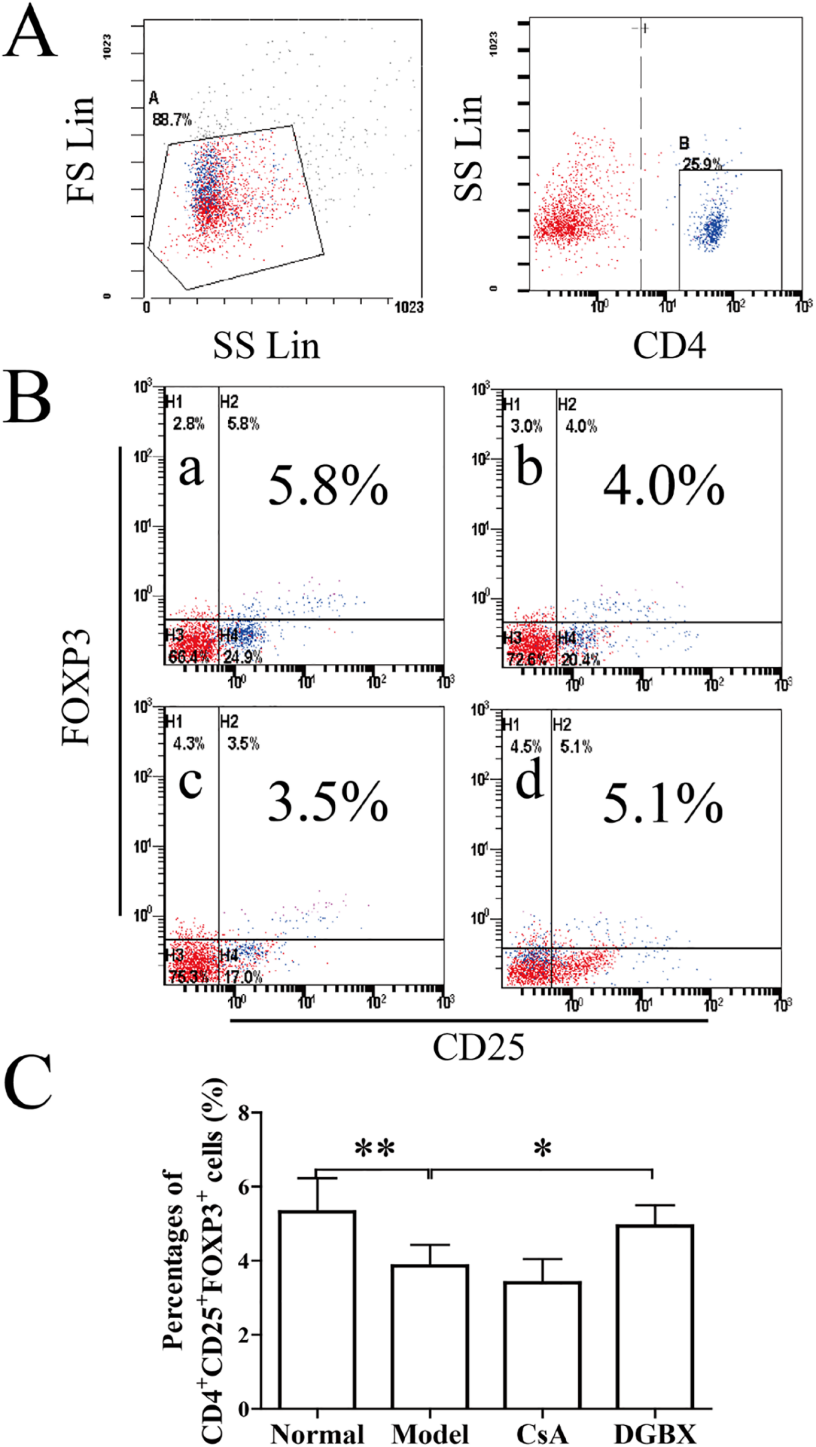
Effect of DGBX on the differentiation of CD4^+^CD25^+^FOXP3^+^ Tregs in splenocytes. (A) The B gate for gating the CD4+ T cells. (B) The percentages of CD4^+^CD25^+^FOXP3^+^ Tregs in splenocytes. (C) The results are presented in the bar charts. a. normal group, b. model group, c. group treated with CsA, and d. group treated with DGBX. Data are presented as means ± SD, n = 6. *P<0.05, **P < 0.01.

The CD4^+^CD25^+^FOXP3^+^ Tregs are indispensable for the maintenance of immunologic self-tolerance and immunosuppression [6, 23]. Impaired function of Tregs has been involved in the pathogenesis of AA [24]. As shown in Fig 5, the percentages of Tregs in AA mice were decreased significantly compared to the normal group (*P*<0.01). However, DGBX increased the differentiation of Tregs markedly compared to the untreated group (*P*<0.05).

### Effects of DGBX on the proliferation and differentiation of Th cells in AA mice

**Fig 6:**
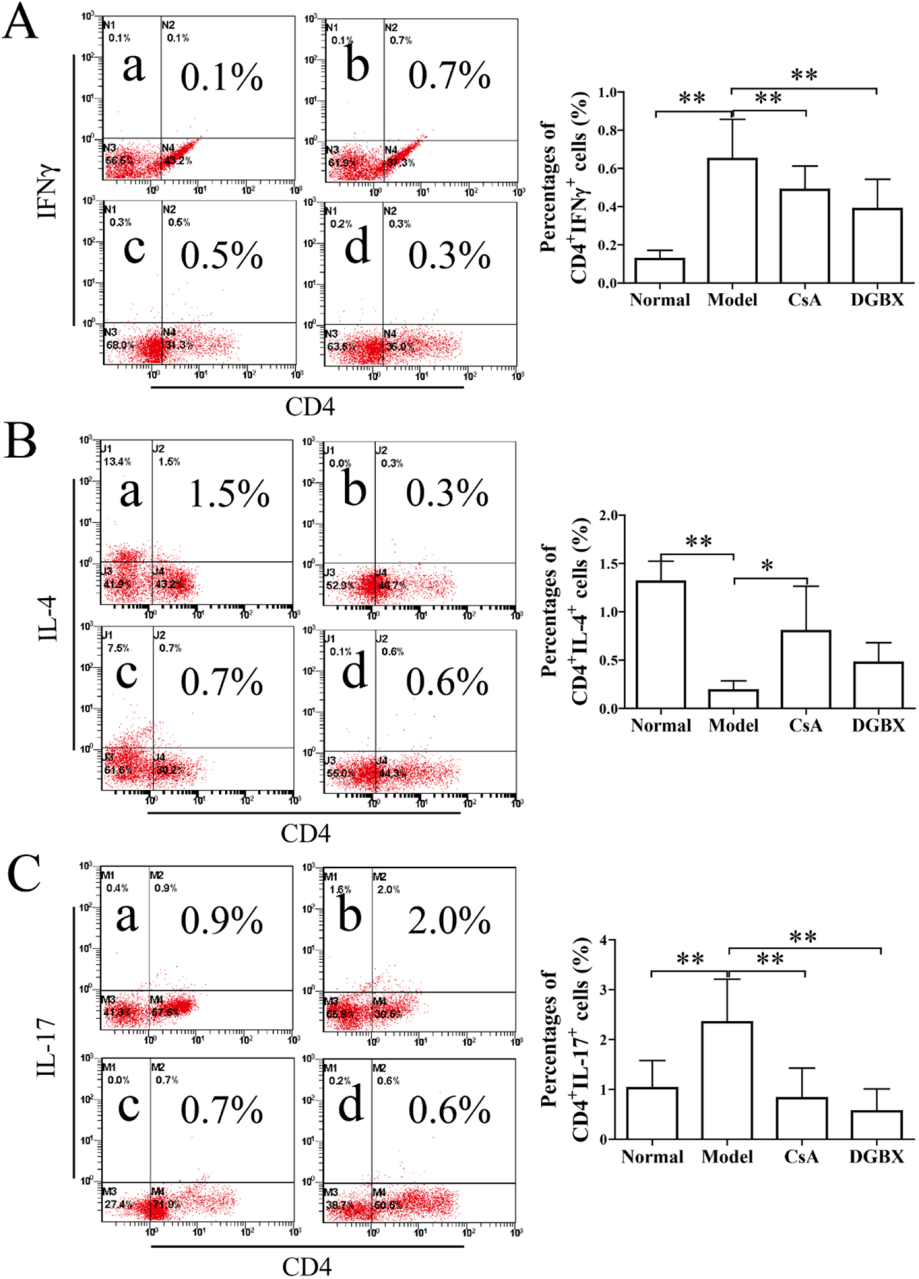
Effect of DGBX on the differentiation of Ths in splenocytes of AA mice. (A) The percentages of CD4^+^IFNγ^+^ Th1 cells. (C) The percentages of CD4^+^IL-17^+^ Th17 cells. The results are presented in the bar charts. a. normal group, b. model group, c. group treated with CsA, and d. group treated with DGBX. Data are presented as means ± SD, n = 6. *P<0.05, **P < 0.01.

A marked expansion of Th1 and Th17 cells and an abnormal phenotype and function of Tregs and Th2 cells are associated with increased severity of AA [25]. Here, we assessed the proliferation and differentiation of Th cells by FACS analysis. As shown in Fig 6, the percentages of CD4^+^IFNγ^+^ Th1 and CD4^+^IL-17^+^ Th17 cells in AA mice were significantly higher than these in normal mice (*P*<0.01), and the level of CD4^+^IL-4^+^ Th2 cells in AA mice was decreased significantly (*P*<0.01, compared with normal group). After four weeks of treatment with DGBX, the abnormally high levels of Th1 and Th17 cells in the splenocytes were down-regulated (*P*<0.01, as compared with model group), but there was no change in the differentiation of Th2 cells. These results indicated that DGBX could regulate the proliferation and differentiation of Th cells in the AA mouse model.

### DGBX decreased the production of inflammatory cytokines in the AA mouse model

**Fig 7:**
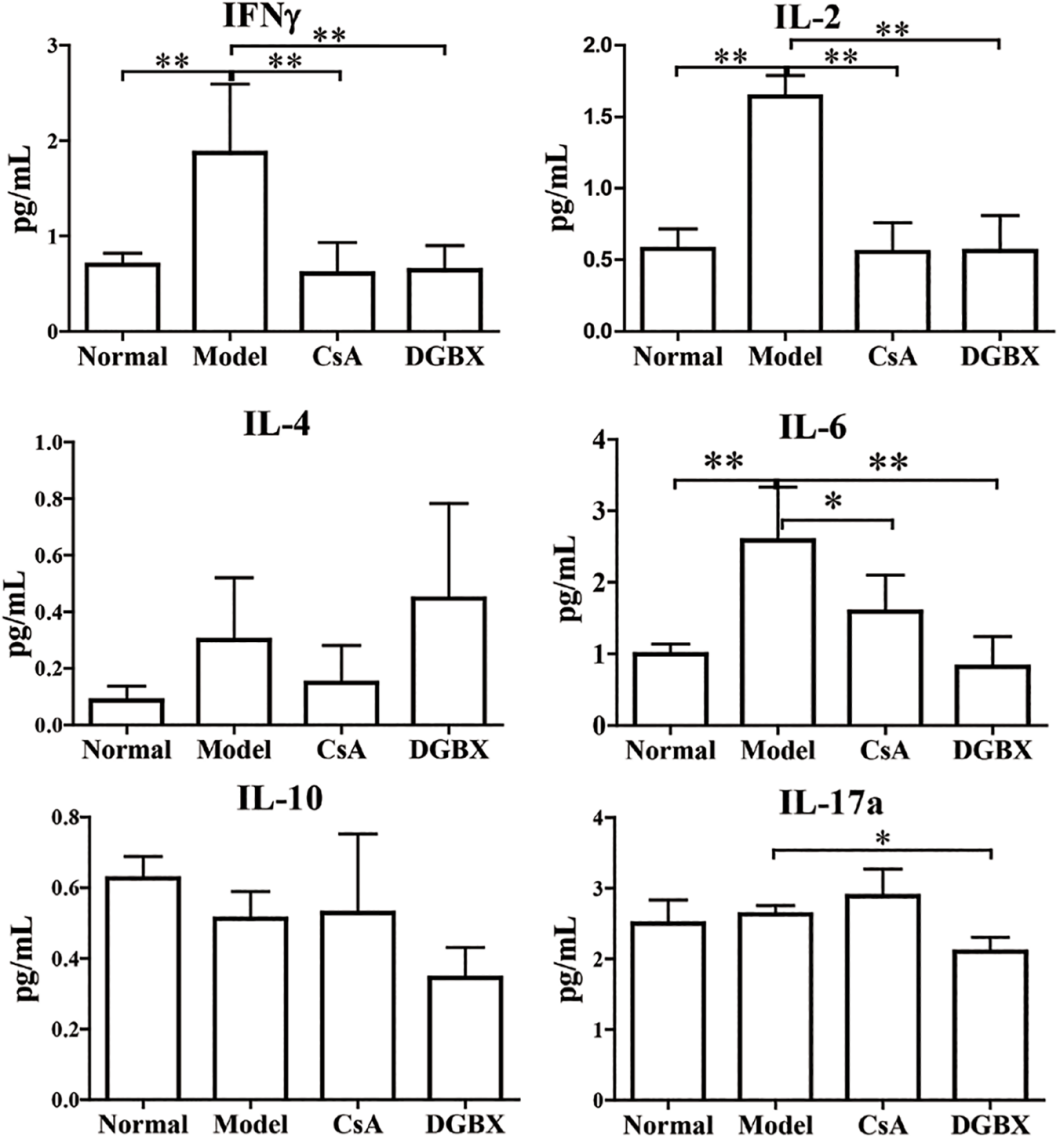
The production of proinflammatory cytokines in the sera of AA mice after treatment evaluation by Bead-based multiplex flow cytometry. The results are presented in the bar charts. Data are presented as means ± SD, n = 6. *P<0.05, **P < 0.01.

We also examined the levels of pro-inflammatory and anti-inflammatory cytokines in sera by bead-based multiplex flow cytometry. The abnormally high levels of pro-inflammatory cytokines, including IL-1β, IL-2, IL-6 and IL-17a, were significantly decreased by DGBX treatment. The quantities of pro-inflammatory cytokines in the treatment group were significantly lower than those in the model group (*P*<0.01 or *P*<0.05). However, DGBX had no effects on the production of anti-inflammatory cytokines (IL-4 and IL-10) (Fig 7).

### DGBX regulated the expression levels of specific transcription factors of Th cells in the AA mouse model

**Fig 8:**
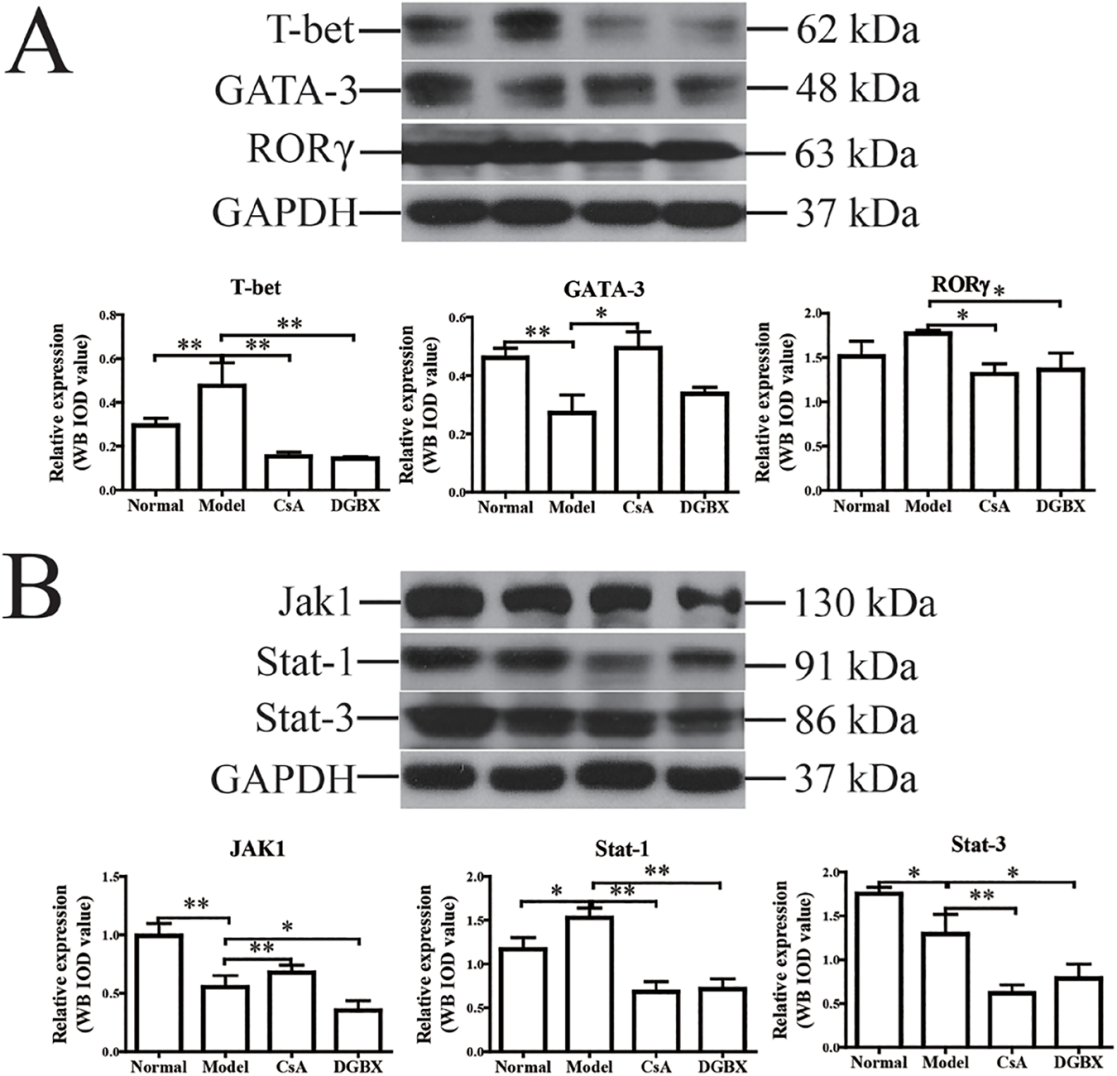
Effects of DGBX on the expression of Th-specific transcription factors and key molecules of the Jak/Stat signaling pathway in AA mice. (A) The expression levels of T-bet, GATA-3 and RORγ were estimated in spleen lysates by western blot analysis. (B) The expressive levels of Jak1, Stat1 and Stat3 were estimated in spleen lysates. GAPDH was used as an internal control. The quantified results are presented in a bar chart. Data are presented as means ± SD, n = 6. *P<0.05, **P < 0.01.

To study the molecular mechanisms by which DGBX regulates the differentiation of Th cells, we assessed the protein levels of Th-specific transcription factors, including T-bet (Th1), GATA-3 (Th2) and RORγ (Th17), by western blot analysis. As shown in Fig 8A, the protein level of T-bet was significantly higher and the level of GATA-3 was lower in the spleen of the AA mouse model (P<0.01), compared with that of normal mice. Treatment with DGBX could significantly down-regulate the expression of T-bet and RORγ in AA mice (*P*<0.05 or *P*<0.01). However, it had no regulatory effect on the expression of GATA-3.

### Effects of XFHM on the expression levels of key molecules of the Jak/ Stat3 signaling pathway

As shown in Fig 8B, the protein levels of key molecules (Jak1, Stat1 and Stat3) of the Jak/Stat signaling pathway in AA mice were significantly decreased by DGBX treatment (*P*<0.01 or *P*<0.05). These results indicated that DGBX could suppress the active immune destruction of hematopoietic cells by inhibiting the activation of the Jak/Stat signaling pathway and by intervening in the expression of key molecules in the Jak/Stat signaling pathway.

## Discussion

AA is an immune-mediated bone marrow failure syndrome. The pathophysiology of AA occurs via immune destruction in HSCs attacked by CD8^+^ cytotoxic T cells (CTLs), CD4^+^ Th1 and Th17 cells [26, 27]. CTLs produce pro-inflammatory cytokines, including IFNγ and TNFα, to activate the Fas-dependent pathway to induce the apoptosis of CD34^+^ HSCs [2]. A proper balance between Th1 and Th2 is important in maintaining the homeostasis of hemopoietic progenitor cells. Untreated AA patients have a higher proportion of Th1 cells, which produce IFNγ and IL-2, due to a shift in the IFNγ/IL-4 ratio induced by the immune response [28]. Aberrant activated Th1 cells also secrete IFNγ and TNFα, contributing to the immune attack of HSCs and inhibiting hematopoietic colony formation [29]. The impaired function of CD4^+^CD25^+^Foxp3^+^ Tregs has been confirmed to contribute to the pathogenesis of autoimmune diseases including AA [30]. Tregs appear to have intrinsic impairment of functions of suppressing normal effector T cell effects and the production of IFNγ in patients with AA [31, 32]. The Treg-mediated immunosuppressive strategy is beneficial to suppress excessive Th immune response in AA [7]. Th17 cells specifically secreting IL-17 were expanded in the acquired AA, which was correlated with the severity of this disease. The expansion of Th17 cells correlated with the depletion of Tregs and showed a reciprocal function between Th17 and Tregs, contributing to the autoimmune process of AA [27]. The combination of Th1 and Th17 expansion and the decrease in the Th2 and Tregs immunophenotype and function contributed to the hematopoietic failure induced by the immune attack on the bone marrow [33].

In the present study, we found that DGBX had regulatory effects on the proliferation and differentiation of T lymphocytes. After 28 days of treatment, DGBX had no effects on the levels of CD3^+^CD8^+^ CTL cells in an immune-mediated AA mouse model. However, the proportion of CD3^+^CD4^+^ T cells was significantly increased by DGBX treatment, which may have been due to the hematosis function of DGBX. Interestingly, DGBX treatment could inhibit the expansion of Th1 and Th17 cells and increase the differentiation of Th2 and Treg cells, for regulating the complex interaction between Th1, Th2, Th17 and Treg in the AA mouse model, thus providing immunosuppressive effects on the immune-mediated bone marrow failure.

The activated signals from the T cell receptor and cytokine receptors initiated the differentiation of effector T lymphocytes. The activation of T cells by IFNγ or IL-4 initiates the differentiation of Th1 and Th2 and then induces the expression of specific transcription factors for driving Th cell differentiation. T-bet and Stat1 contribute to Th1 cells, and GATA-3 specifies Th2 cell differentiation. The transcription factor RORγt is required for Th17 cell specifically [34–36]. In this study, DGBX inhibited the expression levels of T-bet and RORγ and the production of IFNγ, IL-2, IL-6 and IL-17. It also increased the expression of GATA-3 in the AA mouse model. We predicted that DGBX can regulate the proliferation and differentiation of Th cells by intervening in the expression of Th-specific transcription factors and related pro-inflammatory cytokines.

Several studies indicate that the Jak/Stat signaling pathway regulates the functions of cytokines, including erythropoietin, thrombopoietin, and granulocyte colony-stimulating factor during the treatment of anemia [37–39]. IFNγ binding to IFNγ-receptor initiates the IFNγ-induced response [40] and subsequently transduces signals to activate intracytoplasmic Jak tyrosine kinase. Jak tyrosine kinase induces the activation of Stat1, preferentially activates Stat3 phosphorylation, and then translocates signals to the nucleus and induces the transcription of the target genes, contributing to the immune attack and apoptosis in bone marrow cells [41, 42]. Our results showed that DGBX treatment could decrease the expression of key molecules in the Jak/Stat signaling pathway, resulting in immunosuppressive and hematogenic functions of the AA mouse model. These may explain in part the effective mechanism of DGBX treatment for AA.

HPLC-MS has become essential for the analysis of herbal constituents. It is used as a powerful analytical tool for identifying the compounds of herbal formulas in TCM [43, 44]. The base peak spectrum from lyophilized powder of DGBX water decoction was measured to obtain information on its chemical constituents. Eighteen constituents were identified by HPLC-MS analysis. Pharmacological study confirmed that magnoflorine could regulate the differentiation of neutrophils and T cells for anti-inflammation [45]. Coptisine and calycosin could modulate the activation of the nuclear factor-κB (NF-κB), MAPK or PI3K/Akt/GSK-3 signaling pathways, contributing to an inhibitory effect on the inflammatory response induced by pro-inflammatory cytokines [46, 47]. Jatrorrhizine [47], berberine [48] and palmatine [49] have inhibitory functions on cell apoptosis through anti-oxidative activation or intervening in the inducible nitric monoxide synthase system. In this study, the combination of these multiple components had regulatory effects on the proliferation and differentiation of T cells, modulated the activation of the Jak/Stat signaling pathway, and exerted immunosuppressive functions on AA.

## Conclusions

Our sudy confirmed that DGBX treatment could decrease the proliferation and differentiation of effector T cells and impair Treg-mediated immunosuppressive functions. DGBX treatment inhibited the activiation of the Jak/Stat signaling pathway by regulating the expression of key molecules and contributed to the repair of aberrant immune responses and deficiencies in hematopoietic cells. These results suggest that DGBX might be tried in a randomized study for AA, although more data on the possible side effects and doses in humans are needed. The hope is that DGXB could eventually be used against AA in modern complementary and alternative immunosuppressive therapeutics.
